# Frequency and distribution of Notch mutations in tumor cell lines

**DOI:** 10.1186/s12885-015-1278-x

**Published:** 2015-04-25

**Authors:** Anders Peter Mutvei, Erik Fredlund, Urban Lendahl

**Affiliations:** 1 Department of Cell and Molecular Biology, SE-171 77 Stockholm, Sweden; 2 Department of Oncology and Pathology, Science for Life Laboratory, Karolinska Institute, SE-171 77 Stockholm, Sweden

**Keywords:** Signaling pathway, Notch, Cancer, Mutation, p53, Ras, APC, ErbB

## Abstract

**Background:**

Deregulated Notch signaling is linked to a variety of tumors and it is therefore important to learn more about the frequency and distribution of Notch mutations in a tumor context.

**Methods:**

In this report, we use data from the recently developed Cancer Cell Line Encyclopedia to assess the frequency and distribution of Notch mutations in a large panel of cancer cell lines *in silico*.

**Results:**

Our results show that the mutation frequency of Notch receptor and ligand genes is at par with that for established oncogenes and higher than for a set of house-keeping genes. Mutations were found across all four Notch receptor genes, but with notable differences between protein domains, mutations were for example more prevalent in the regions encoding the LNR and PEST domains in the Notch intracellular domain. Furthermore, an *in silico* estimation of functional impact showed that deleterious mutations cluster to the ligand-binding and the intracellular domains of NOTCH1. For most cell line groups, the mutation frequency of Notch genes is higher than in associated primary tumors.

**Conclusions:**

Our results shed new light on the spectrum of Notch mutations after *in vitro* culturing of tumor cells. The higher mutation frequency in tumor cell lines indicates that Notch mutations are associated with a growth advantage *in vitro*, and thus may be considered to be driver mutations in a tumor cell line context.

**Electronic supplementary material:**

The online version of this article (doi:10.1186/s12885-015-1278-x) contains supplementary material, which is available to authorized users.

## Background

Our understanding of the molecular basis for cancer is rapidly improving, to a large extent owing to the recent progress in DNA sequencing technologies, which now allows the mutational landscape to be explored in a genome-wide manner both in primary tumors and in tumor cell lines [1]. Thanks to these efforts, it is becoming increasingly apparent that there are a small number of very frequently mutated genes, along with a longer “tail” of genes with fewer mutations. A recent insight is also that tumors are endowed with specific sets of mutational signatures that shed light on their history, both with regard to internal processes such as defective DNA repair, but also reflecting external processes, such as exposure of cells to ultraviolet light or tobacco smoking [2]. By analyzing the distribution of mutations in individual mutated genes, oncogenes and tumor suppressor genes can increasingly be identified and our understanding of which mutations that are driver mutations, i.e. a mutation that confers a growth advantage for the tumor, and passenger mutations, i.e. mutations bringing no selective advantage to the tumor, is rapidly improving. It is an emerging view that a relatively limited set of evolutionarily conserved signaling pathways harbor the majority of driver mutations and this list includes for example the PI3K, MAPK, Hedgehog and, important for this study, the Notch signaling pathway [1]. However, the relationship between a particular signaling pathway and tumor development is still rather unexplored, and better insights into the mutational spectrum will be important for understanding how deregulation of a signaling pathway contributes to cancer and, in the long-term, for future therapy development.

To gain further insights into the link between Notch signaling and tumor development, we have analyzed the extent of Notch mutations in tumor cell lines, using information from the recently published Cancer Cell Line Encyclopedia (CCLE), which contains deep genomics and transcriptome information for more than 900 cell lines with pharmacological profiles for a range of cancer therapeutics [3]. Notch signaling is a highly conserved cell-cell interaction mechanism with a relatively simple molecular architecture but a diverse, cell context-specific, signaling output [4,5]. Notch signaling is initiated when a transmembrane Notch receptor is activated by transmembrane ligands, of the Jagged or Delta-like type, on neighboring cells. Ligand activation of the Notch receptor leads to proteolytic cleavage of the receptor and release of its intracellular domain (Notch ICD). Notch ICD relocates from the membrane to the nucleus and binds to the DNA-binding protein CSL (also referred to as RBP-Jκ or CBF1), leading to activation of Notch downstream genes [5,6].

There are a number of links between deregulated Notch signaling and cancer. Direct mutations or copy number variations are observed in acute lymphoblastic T-cell leukemia (T-ALL), non-small cell lung cancer (NSCLC) and, to a lesser extent, breast cancer [7-9]. Furthermore, deregulation of Notch signaling is observed in a broad range of tumors. For example, in breast cancer, increased Notch signaling, in the form of high Jagged1 expression, is frequently observed [10,11]. On the other hand, and in keeping with the cell context-dependent signaling output, Notch can also act as a tumor suppressor gene. In the skin, Notch signaling promotes, rather than blocks, differentiation [12] and in line with this, Notch mutations in squamous cell carcinoma (SCC) are usually inactivating [13-15]. With these multiple links to cancer, it is not surprising that development of Notch therapies is a very active research area and although there are yet no clinically approved therapies, there are ongoing clinical trials for a number of indications [6].

In this report, we ask a number of questions regarding the frequency of Notch mutations in tumor cell lines and primary tumors: How frequent are Notch mutations in tumor cell lines as compared to other well-established oncogenes and house-keeping genes? How are mutations distributed across the various Notch receptors? Is there a preference for particular mutations in specific tumor cell line types? Our data indicate that Notch mutations occur at a frequency that suggests that they may confer growth advantage during *in vitro* culture, i.e. that they would be driver mutations, and we also identify receptor-specific patterns of mutations. Information regarding the spectrum of mutations to Notch receptors in cancer cell line models can be a valuable resource for future Notch research and may aid in the development of Notch targeted therapies in cancer.

## Methods

The CCLE dataset was downloaded from the CCLE-database (http://www.broadinstitute.org/ccle). The dataset was generated using a hybrid capturing assay together with massively parallel sequencing and contains a list of mutation and indels in 1651 genes across 905 cancer cell lines, aligned to the human genome assembly hg19, where the following variants had been filtered out: common polymorphisms, allelic fractions below 10%, putative neutral variants and mutations located outside the coding DNA sequence. Mutations in introns, a (CTG)_*n*_-Notch4 polymorphism in exon 1 [16], non-synonomous mutations and cell types having less than 5 cell lines were also filtered out. The cell type barcodes “haematopoietic_and_lymphoid_tissue” and “skin” that are used in the CCLE-dataset are referred to as “blood” and “melanoma”, respectively, throughout this manuscript.

Datasets kept on the cBioPortal server were used for computing mutational frequencies in primary tumors, utilizing the CGDS-R package in R (http://www.R-project.org) [17,18]. The following datasets were utilized for the analyses (totaling more than 2900 tumors; the sizes of the data sets used are indicated in parenthesis): Endometrial cancer (n = 248) [19], prostate cancer (n = 112) [20], large intestine (n = 224) [21], esophageal cancer (n = 146) [22], lung cancer (n = 230) [23], glioblastoma (n = 291) [24], ovarian cancer (n = 316) [25], skin cancer (n = 121) [26], liver cancer (n = 231) [27], pancreatic cancer (n = 99) [28], breast cancer (n = 507) [29] and renal cancer (n = 424) [30]. The study was conducted in accordance with the ethical guide lines from the Central Ethical Review Board in Sweden, as of June 1, 2008.

The CGDS-R package was utilized to explore putative copy number alterations of Notch receptor genes in the CCLE data set. The Cbioportal MutationMapper online tool (http://www.cbioportal.org/public-portal/mutation_mapper.jsp) was used for generating supplementary mutation distribution plots. All other analyses were performed using R. In Figure 2E, the SCC cell lines used in the analysis are: BICR56, TE11, OE21, TE8, KYSE270, KYSE180 and KYSE450.

### Calculation of mutation frequency

**Figure 1:**
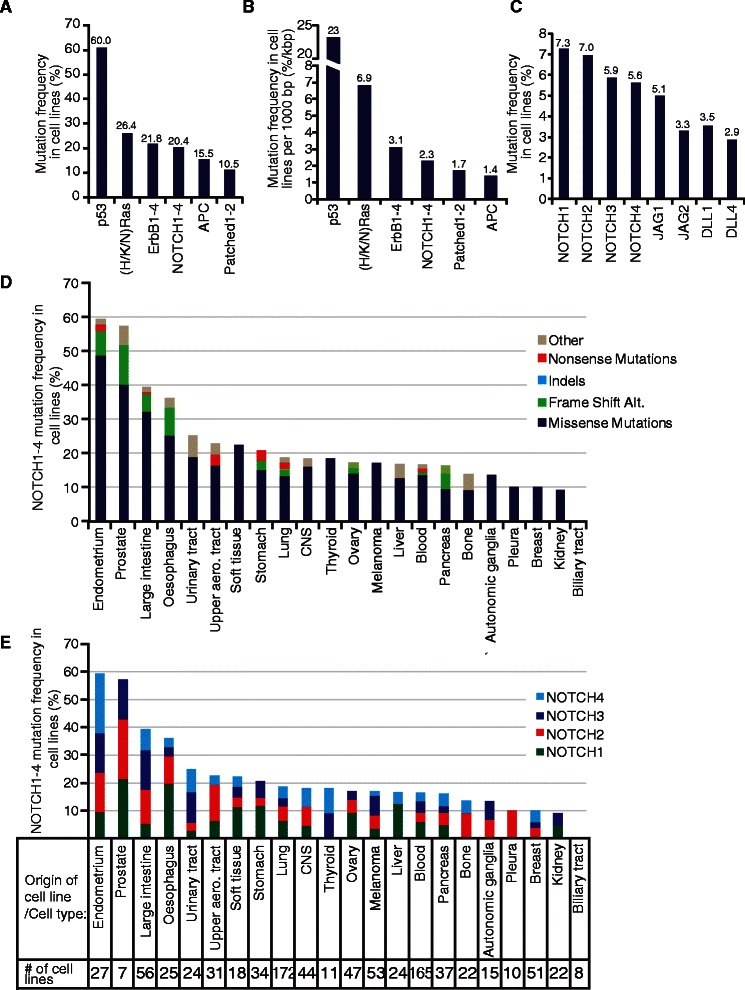
Notch components are frequently mutated in established cancer cell lines. **(A**-**B)** Overall mutation frequency (percentage mutated cell lines) of NOTCH receptors 1–4, as well as of the three Ras proteins (H/K/N-Ras), ErbB1-4, p53, Patched1-2 and APC, as indicated. In **(B)**, the mutation frequencies are relative to the average coding region size (%/kbp). **(C)** Mutation frequency of Notch receptors and ligands. **(D**-**E)** Mutation frequency of NOTCH receptors 1–4 in cell lines that have been clustered into specific cell types, as indicated. In **(D)**, the relative distribution of the different types of NOTCH receptor mutations is plotted for each cell type. In **(E)**, the relative distribution of mutations in each NOTCH receptor is plotted for each cell type. The number (#) of cell lines for each cell type is specified in **(E)**. Indels = Insertions or deletions (not causing frame shift alterations). Frame Shift Alt = Frame Shift Alterations. bp = base pairs. In **(A**-**C)**, the mutation frequency for the corresponding gene/genes is indicated above each bar.

The mutation frequency was calculated as the number of mutations in a specific gene or gene family across all cell lines or across a specific cell type. The CCLE classification of cell lines into different cell types, as specified in the CCLE-dataset, was used. When determining mutation frequencies for gene families with multiple genes (e.g. Notch 1–4 or H/K/N-Ras), maximum one mutation per cell line was counted. When determining the relative distributions of mutational types in Figure 1D and Notch receptors in Figure 1E, all mutations were taken into account. The Protein ANalysis THrough Evolutionary Relationships (PANTHER; http://www.pantherdb.org/) online cSNP tool was used to estimate the impact of missense mutations on protein function, using data from PANTHER version 6.1 [31,32].

## Results

### The mutation frequency of Notch receptors and ligands compared to other oncogenes and tumor suppressor genes

In CCLE, more than 1,600 genes, including most known oncogenes and tumor suppressor genes, have been sequenced across more than 900 human tumor cell lines [3]. CCLE is therefore an ideal resource to explore mutation patterns and frequencies in a large scale. To gain new insights into Notch mutations, we first asked how the frequency of Notch mutations compared to the mutation frequencies in other well-established oncogenes (H, K and N (H/K/N) Ras and ErbB1-4) and tumor suppressor genes (p53, APC and Patched1-2). The mutation frequency for p53 was highest (60.0%), followed by the combined score for H/K/N Ras genes (26.4%), ErbB genes (21.8%), NOTCH1-4 (20.4%), APC (15.5%) and Patched1-2 (10.5%) (Figure 1A). Since a larger coding region on average is more prone to accumulating mutations, we also recalculated the data in Figure 1A relative to the size of the coding regions. This reveals that the p53 and Ras family showed considerably higher mutation frequencies than the other genes, but the Notch mutation frequency was in the same range as for the ErbB1-4, Patched1-2 and APC genes (Figure 1B). The high scores for p53 and Ras likely reflect the exceptional selective advantage of mutations in these two genes for tumor growth and during in vitro establishment of cell line cultures. When the four different Notch receptors and four of the ligands (JAG1, JAG2, DLL1 and DLL4) were analyzed individually, we found that the mutation frequency for the genes ranged from 7.3% (NOTCH1) to 2.9% (DLL4) (Figure 1C).

We next asked whether the frequency of Notch receptor gene mutations differed in cell lines derived from different tumor types. The data show that the mutation frequency was highest in cell lines from endometrial, prostate and large intestine tumors (Figure 1D), which also were the cell types that harbored most mutations overall (Additional file 1: Figure S1A). Cell lines from the oesophagus and urinary tract showed a somewhat lower mutation frequency, but in all cases higher than 25% (Figure 1D). At the other end of the spectrum, a much lower Notch mutation frequency was noted in tumor cell lines from mesothelioma (pleura), breast and kidney (less than 10%), and with no mutations in tumors from the biliary tract (Figure 1D). 5.0 % of the cell lines carried more than one mutation in NOTCH1-4 (data not shown).

We next analyzed what types of Notch receptor gene mutations were most prevalent in the tumor cell lines. Across all tumor types, missense mutations were by far the most dominant category, with a smaller proportion of frame shift alterations and nonsense mutations and with only a very small proportion of indels (insertions/deletions) (Figure 1D). While Notch is a tumor suppressor gene in skin [13-15], we did not find any nonsense mutations in tumor cell lines derived from melanomas. Finally, we assessed whether mutations in a specific Notch receptor gene were associated with a particular tumor cell line type. In the majority of the tumor cell line types, mutations were found in at least three different Notch receptor genes, but with liver as a notable exception, where only NOTCH1 and NOTCH4 were found to be mutated (Figure 1E).

### The distribution of mutations in the four Notch receptor genes

**Figure 2:**
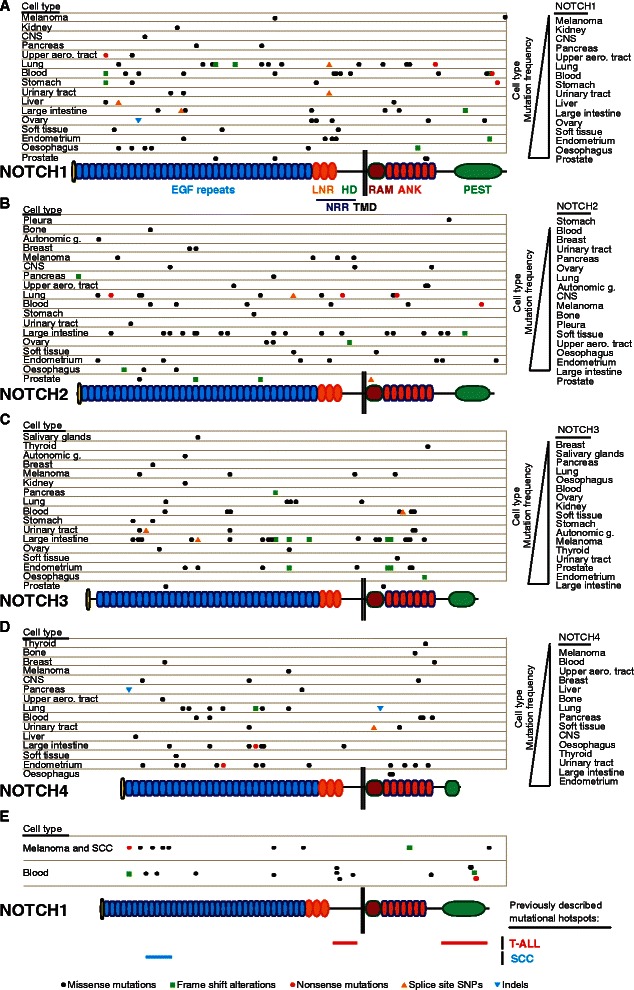
Notch receptor mutation spectra for different cancer cell types. (**A**-**E**, left side) Mutation spectra for NOTCH1 **(A**,**E)**, NOTCH2 **(B)**, NOTCH3 **(C)** and NOTCH4 **(D)**. (**A**-**D**, right side) Cell types ordered after mutation frequency for the corresponding Notch receptor. The Notch receptor domains are listed in **(A)**. In **(E)**, previously described mutational hotspots in T-ALL and SCC are marked. LNR = Lin12-Notch repeats, HD = heterodimerization domain, NRR = negative regulatory region, RAM = RBPJκ associated module, ANK = ankyrin repeats, PEST = proline, glutamic acid, serine and threonine rich domain. Indels = Insertions or deletions, Frame Shift Alt = Frame Shift Alterations. Upper aero. tract = Upper aerodigestive tract. Autonomic g. = autonomic ganglia. CNS = central nervous system. The different types of mutations are described at the bottom of the figure. Only mutations in the coding region of the Notch receptor proteins are shown.

The CCLE data set also allowed us to analyze the distribution of mutations across the four different Notch receptor genes, to learn whether there are mutational hotspots or whether a particular type of mutation associates with a particular receptor. Mutations were largely scattered along the length of the receptors (Figure 2, left hand side; Additional file 1: Figure S1 B-E), with one exception: a frame shift alteration in NOTCH3 clustered at amino acid position 1802 (Figure 2C; Additional file 1: Figure S1D). Missense mutations were the most common form of mutations for all Notch receptor types, followed by frame shift alterations for NOTCH1-3; however, this class of mutations was not present to the same extent in NOTCH4 (Figure 2; Additional file 1: Figure S1F). More than 50% of the mutations resided in the EGF repeat region of each receptor (NOTCH1 = 51.3%, NOTCH2 = 59.72%, NOTCH3 = 58.5% and NOTCH4 = 66.0%). Missense mutations involving a cysteine residue (either gain or loss) in the EGF repeats is a hallmark for CADASIL mutations in NOTCH3 [33], but we did not find any missense mutations affecting cysteine residues in the CCLE data set (Figure 2C). Missense mutations were also the most common form of mutation in Notch ligands (Additional file 1: Figure S2).

Gain-of-function mutations in T-ALL, NSCLC and breast cancer cluster in the negative regulatory region (NRR) and PEST domains (Figure 2E) [7-9,34], and we therefore analyzed whether mutations were more prevalent in these regions and whether they differed between different receptors. PEST domain mutations were frequently observed in NOTCH1, and two mutations were found in NOTCH2, whereas there were no such mutations in NOTCH3 and NOTCH4 (Figure 2A-D). For the NRR region, which encompasses the LNR region and heterodimerization domain (HD), we found a mutation spectrum similar to the PEST domain, i.e. only a few mutations were found in NOTCH3 or NOTCH4, whereas 21.1% of the NOTCH1 mutations and 12.2% of the NOTCH2 mutations resided in this region (Figure 2A-D). Prostate was the most frequently mutated cell type for both NOTCH1 and NOTCH2 but prostate tumor cell lines did not contain any mutations in NOTCH3 or NOTCH4 (Figure 2A-D, right hand side). To learn more about the differences in the mutational pattern between skin and leukemias, which harbor gain- and loss-of-function Notch mutations, respectively [7,12], we derived the NOTCH1 mutational pattern from blood cell lines, including T-ALL, and compared with cell lines derived from melanoma and SCC. Approximately half of the mutations from blood cell lines were found in the NRR and PEST domains, in contrast to melanoma and SCC, where only one mutation were found in these two domains (12.5%; Figure 2E).

**Figure 3:**
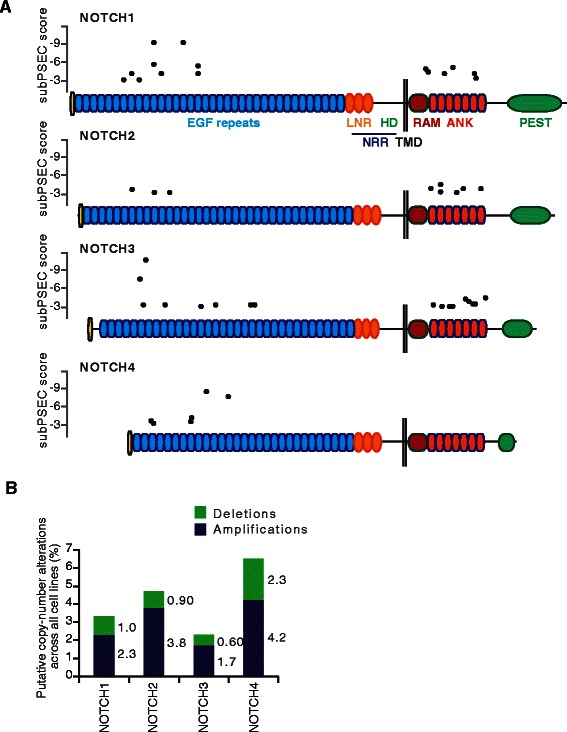
Copy-number alterations and estimation of the impact of missense mutations on Notch1-4 receptor function using PANTHER cSNP. **(A)** Schematic illustrations of the NOTCH1-4 receptors, showing the distribution of missense mutations with a subPSEC score lower than −3, i.e. 50% chance to be deleterious. The abbreviations for the Notch receptor domains are explained in the legend of Figure 2. **(B)** The distribution of deletions and amplifications in the four Notch receptors is shown, with the percentage written to the right of the corresponding bar.

To explore the potential functional impact of the Notch receptor mutations, we scored all missense mutations for NOTCH1-4 with subPSEC (**sub**stitution **p**osition-**s**pecific **e**volutionary **c**onservation), using the Protein ANalysis THrough Evolutionary Relationships (PANTHER) cSNP tool. The subPSEC score is an estimate of the likelihood for a given non-synonymous mutation to functionally impact on the protein, ranging from 0 (neutral) to −10 (the highest likelihood to be deleterious), and a subPSEC score below −3 is regarded as a cutoff for functional significance [31,32]. 47 of the 215 missense mutations in NOTCH1-4 (~22%) were estimated to have a deleterious effect on the proteins, i.e. with a score between −3 and −10. Interestingly, the mutations with a subPSEC below −3 clustered in the ankyrin domain and, for NOTCH1, in EGF repeats 11 and 15, a portion of the receptor containing the ligand-binding domain [35,36], as well as in the ICD (Figure 3A; Additional file 2: Data 1).

Finally, since Notch receptors have been reported to be amplified in ovarian cancers [37], we investigated if the Notch receptors were subjected to copy number alterations in cell lines. We utilized the CGDS-R cBioPortal package for R to obtain data on putative copy-number alterations, which have been computed using the GISTIC2 algorithm [17,18,38]. Between 1.7% and 4.2% of all cell lines had Notch high level amplifications, Notch4 being the most frequently amplified (Figure 3B). Deletions were less common for all Notch receptors, ranging from 0.6% to 2.3% (Figure 3B).

### The frequency of Notch mutations in tumor cell lines as compared to primary tumors

Next, we compared the Notch mutation frequency in tumor cell lines with the frequency observed in primary tumors for the corresponding organs, using several data sets kept on the cBioPortal server, the majority derived from The Cancer Genome Atlas (TCGA). We reasoned that this could serve as an estimate of whether mutations accumulate over time during the culturing of tumor cell lines, which in turn may indicate whether such mutations offer a growth advantage *in vitro*. To better relate Notch to other gene categories, we compared mutation frequencies for Notch components to the set of oncogenes and tumor suppressor genes used in Figure 1A-B (p53, H/K/N Ras, APC, Patched1-2 and ErbB1-4) as well as to a set of house-keeping genes that have not been reported to be involved in tumor formation: polyadenylate-binding nuclear protein1 (PABPN1), fucose-1-phosphate guanylyltransferase (FPGT) and Non-POU domain-containing octamer-binding protein (NONO) [39]. PABN1 encodes a protein involved in mRNA polyadenylation [40], FPGT encodes a metabolic enzyme [41] and the RNA-binding protein NONO was recently implicated in the regulation of the circadian clock [42].

**Figure 4:**
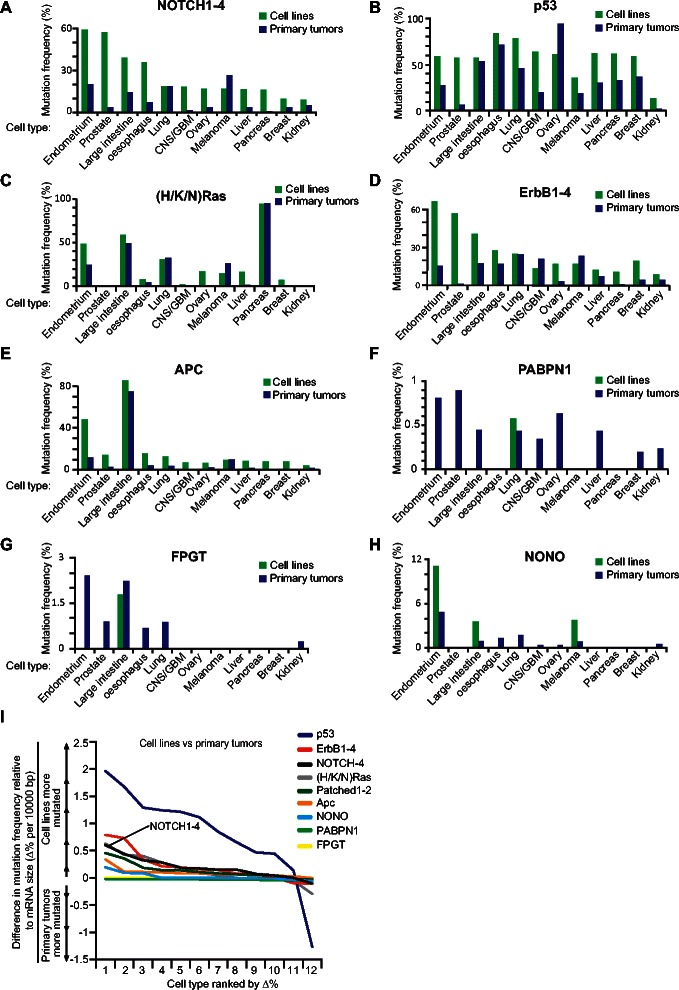
Notch receptors constitute mutational hot spots in cancer cell lines. **(A**-**H)** Mutation frequencies of NOTCH1-4 **(A)** and proteins that are well known in the pathology of cancer **(B**-**E)**, as well as house-keeping proteins that do not have an established role in tumor formation **(F**-**H)**. Mutation frequencies are shown for both cancer cell lines and primary tumors for 12 different cell types, as indicated. In **(I)**, the difference in percentage (∆%) between cell lines and primary tumors for each protein/protein family in **(A**-**H)**, and for each cell type have been ranked and plotted. Values have been normalized to the average coding region size of each protein/protein family (∆%/10000 bp).

Notch mutation frequencies were found to be higher in tumor cell lines than in primary tumors for all cell types except for lung, which had the same frequency, and melanoma, which had a higher mutation frequency in primary tumors (Figure 4A). The largest differences in frequency were observed for the endometrium and prostate cell types (Figure 4A) which however also had the largest accumulation of mutations *in vitro* overall (Additional file 1: Figure S1A). A similar increase in mutation frequency in tumor cell lines was found in the majority of cell types for APC, p53, Patched1-2 and ErbB1-4 (Figure 4B,D,E,I; Additional file 1: Figure S3A,D,E). H/K/N Ras, on the other hand, showed a more complex pattern with an increase in endometrium ovary, liver, large intestine and breast, but not in the other tumor types (Figure 4C). Notch ligands (JAG1-2, DLL1,4), like Notch receptors, showed higher mutations frequencies in tumor cell lines, although these were mainly restricted to the endometrium and prostate cell types (Additional file 1: Figure S3B,C). In contrast, mutation frequencies were overall very low for the house-keeping genes, with lower frequencies in tumor cell lines compared to primary tumors across almost all cell types (Figure 4F,G,H,I; Additional file 1: Figure S3D-E). In sum, these data suggest that tumor cell lines generally contain a higher number of mutations in established oncogenes and tumor suppressors compared to corresponding primary tumors. This notion holds true also for Notch receptors, and to some extent Notch ligands, but not for the house-keeping genes.

## Discussion

There is an emerging view that deregulated Notch signaling is linked to cancer and this notion receives support both from the identification of specific mutation patterns in Notch receptors, as well as from numerous studies reporting altered Notch signaling levels in a broad set of tumor types. In keeping with a cell context-specific signaling output, Notch can act as an oncogene or tumor suppressor gene, depending on the tissue of origin. These multi-faceted links between Notch and cancer prompted us to address to what extent Notch genes are mutated in established tumor cell lines, as such information would be a valuable resource to better understand Notch signaling and its role in the control of cellular growth *in vitro*. An important conclusion from our data is that the mutational frequency for the Notch receptors was similar to that of the well-established oncogenes ErbB1-4 and the tumor suppressor genes Patched1-2 and APC, whereas H/K/N Ras and p53, as expected, showed considerably higher frequencies. Furthermore, the frequency of Notch mutations was higher in tumor cell lines when compared to primary tumors, which was also the case for the majority of the other oncogenes and tumor suppressor genes, but not for a set of house-keeping genes. Although mutations might be more easily detectable in cell lines because of their homogenous nature, the substantial increase in mutation frequency argues that Notch mutations become enriched in multiple tumor cell lines during *in vitro* culturing. Notch mutations may thus confer a growth advantage and could be considered to be driver mutations for *in vitro* growth, although this remains to be functionally tested in future studies. It should also be kept in mind that accumulation of mutations in cell lines may not be completely linked to growth advantages, as primary tumors rarely are completely pure, but may be contaminated with stromal cells. Moreover, mutations in CCLE, in contrast to TCGA, contains private germline variants [43]. The hypothesis that at least some of the Notch mutations may be driver mutations is of interest from a therapeutic perspective. Considerable efforts are made to develop novel therapies that blocks or ameliorates Notch signaling, with several strategies currently being evaluated in preclinical and clinical trials [6]. It would be interesting to functionally test mutations identified in this study, to learn if there are novel uncharacterized gain-of-function mutations which could serve as future therapeutic targets.

The mutations in the Notch receptor genes were predominantly missense mutations, which is in keeping with the overall mutation spectrum in tumors [1]. Gene loss frequency for the Notch receptor genes was in contrast rather low. The Notch mutations were distributed along the length of the four Notch receptor genes, a distribution also observed for NOTCH1 in a smaller breast cancer data set [44]. In the EGF repeat domain at the extracellular side, mutations were found across the majority of the EGF repeats, but none of the mutations led to a gain or loss of a cysteine residue, which is the defining mutation for NOTCH3 in CADASIL [33]. CADASIL NOTCH3 mutations are however not considered to provide a growth advantage, but rather to lead to degeneration of vascular smooth muscle cells. Interestingly, we identified receptor-specific differences in the mutation spectrum, in particular for the NRR region and the PEST domain in the intracellular domains, where NRR and PEST mutations were more prevalent in NOTCH1 and 2, as compared to NOTCH3 and 4. The receptor bias for PEST mutations is interesting given the important role of the PEST domain in regulating the stability of Notch ICD. NOTCH1 PEST domain mutations are frequently observed in T-ALL, where they are gain-of-function mutations leading to a more long-lived form of NOTCH1 ICD [7,45]. When mutations were scored for potential impact on protein function, with a subPSEC score below −3, most NOTCH1 mutations clustered in the ligand-binding region or in the ICD. Moreover, in keeping with the tumor suppressor role of NOTCH1 in skin [12], it is of note that melanoma cancer cell lines contained the fewest NOTCH1 mutations and melanoma was also the only cell type that had a higher mutation frequency in primary tumors compared to cell lines.

The catalog of Notch ICD mutations generated by our analysis of the CCLE data base provides an opportunity to functionally characterize the effects of these mutations, for example with regard to alterations in signaling strength [46], intracellular routing [47] or the ability to intersect with other signaling mechanisms [6]. The Notch ICD serves as an interaction hub for the cross-talk between Notch signaling and other signaling mechanisms such as the cellular hypoxic response and the BMP/TGF-beta signaling pathway, where Notch ICD interacts with HIF and SMAD proteins, respectively (for review see [5]). Notch also cross-talks with PI3K and NF-kB signaling [4,48], and to learn if the mutations affect these cross-talks is of interest from the perspective of Notch therapy development.

## Conclusions

The mutation frequency of Notch receptor genes in established tumor cell lines is similar to that of established oncogenes and tumor suppressors. Moreover, Notch mutations are found at a higher frequency in tumor cell lines compared to primary tumors. This implies that Notch mutations may be connected with a growth advantage *in vitro* and thus may be considered to be driver mutations in tumor cell lines.

## Additional files

Additional file 1: Figure S1. **(A)** The overall number of mutations per cell line for the different cell types. **(B-E)** Lollipop plots to visualize potential clustering of mutations for NOTCH1 **(B)**, NOTCH2 **(C),** NOTCH3 **(D)** and NOTCH4 **(E)**. The color of the pins denote the different mutation types in the following way: black = indels, green = missense mutations, red = truncating mutations (nonsense, nonstop, frameshift alterations and splice site alterations), gray = other mutations. **(F)** The overall number of the different types of mutations across the four Notch receptor genes. LNR = Lin12-Notch repeats, ANK = ankyrin repeats, PEST = proline, glutamic acid, serine and threonine rich domain. **Figure S2.** Mutations in Notch ligands in the CCLE data set **(A)** Lollipop plots to visualize mutations for JAG1, JAG2, DLL1 and DLL4. The color code of the pins is explained in the legend of Supplementary Figure 1. MNNL = N-terminal domain of Notch ligands, DSL = Delta-Serrate-LAG-2 domain **(B)** The overall number of the different types of mutations across four of the Notch ligand genes. The transmembrane domain is denoted by a vertical bar. **Figure S3.** Notch receptors constitute mutational hot spots in established cancer cell lines. **(A-C)** Mutation frequencies of Patched1-2 **(A)**, Jagged1 and 2 **(B)**, and Delta-like 1 and 4 **(C)**. **(D-E)** The differences in percentage (∆%) between cell lines and primary tumors for each protein/protein family in Figure 4A-H and Supplementary Figure 3A for each cell type have been ranked and plotted (without normalization to the average coding region size of each protein/protein family, which is shown in Figure 4I).
Additional file 2: **Data 1.** Notch receptor and ligand mutations in different cell lines, as specified in the CCLE-dataset.

## Abbreviations

CCLE: Cancer Cell Line Encyclopedia
TCGA: The Cancer Genome Atlas
ICD: Intracellular domain
NICD: Notch intracellular domain
T-ALL: T-cell acute lymphoblastic leukemia
NSCLC: Non-small cell lung cancer
PANTHER: The Protein ANalysis THrough Evolutionary Relationships
Indels: Insertions/deletions
NRR: Negative regulatory region
HD: Heterodimerization domain
subPSEC: Substitution position-specific evolutionary conservation
FPGT: Fucose-1-phosphate guanylyltransferase
NONO: Non-POU domain-containing octamer-binding protein
PABPN1: Polyadenylate-binding nuclear protein1
