# Supplementary material for: Frequency and distribution of Notch mutations in tumor cell lines
Source: BMC Cancer. 2015 Apr 25;15:311. doi: 10.1186/s12885-015-1278-x (PMC4430925; doi:10.1186/s12885-015-1278-x)
Supplement: Additional file 1: Figure S1. — (A) The overall number of mutations per cell line for the different cell types. (B-E) Lollipop plots to visualize potential clustering of mutations for NOTCH1 (B), NOTCH2 (C), NOTCH3 (D) and NOTCH4 (E). The color of the pins denote the different mutation types in the following way: black = indels, green = missense mutations, red = truncating mutations (nonsense, nonstop, frameshift alterations and splice site alterations), gray = other mutations. (F) The overall number of the different types of mutations across the four Notch receptor genes. LNR = Lin12-Notch repeats, ANK = ankyrin repeats, PEST = proline, glutamic acid, serine and threonine rich domain. Figure S2. Mutations in Notch ligands in the CCLE data set (A) Lollipop plots to visualize mutations for JAG1, JAG2, DLL1 and DLL4. The color code of the pins is explained in the legend of Supplementary Figure 1. MNNL = N-terminal domain of Notch ligands, DSL = Delta-Serrate-LAG-2 domain (B) The overall number of the different types of mutations across four of the Notch ligand genes. The transmembrane domain is denoted by a vertical bar. Figure S3. Notch receptors constitute mutational hot spots in established cancer cell lines. (A-C) Mutation frequencies of Patched1-2 (A), Jagged1 and 2 (B), and Delta-like 1 and 4 (C). (D-E) The differences in percentage (∆%) between cell lines and primary tumors for each protein/protein family in Figure 4A-H and Supplementary Figure 3A for each cell type have been ranked and plotted (without normalization to the average coding region size of each protein/protein family, which is shown in Figure 4I). [file 12885_2015_1278_MOESM1_ESM.pdf]

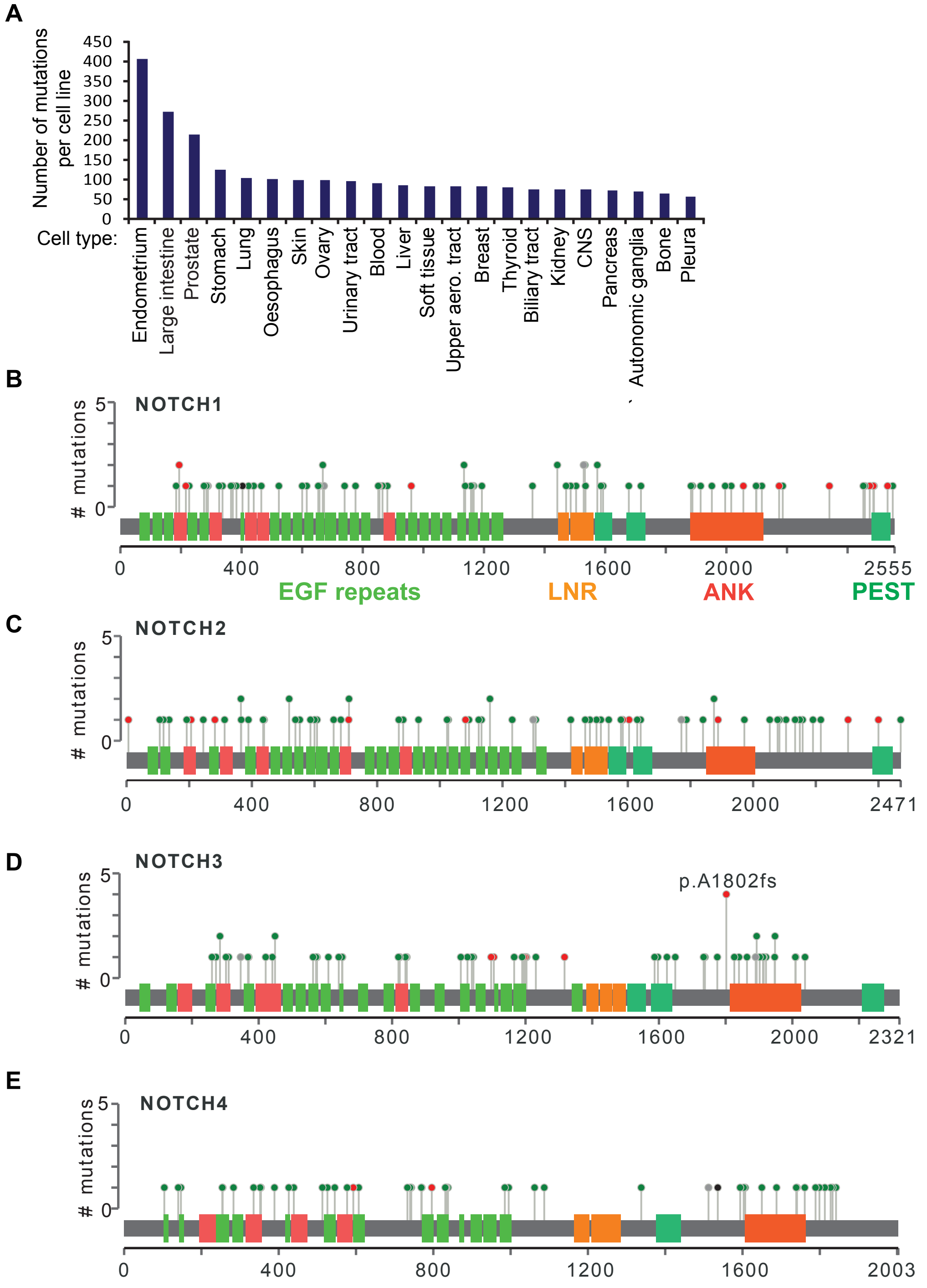

F

Mutational type and number of mutations for each Notch receptor					
#	Missense mutations	Frame shift alterations	Nonsense mutations	Indels	Splice Site SNP
NOTCH1	59	8	4	1	4
NOTCH2	60	6	4	0	2
NOTCH3	52	10	0	0	3
NOTCH4	44	1	2	2	1

Supplementary Figure 1

A

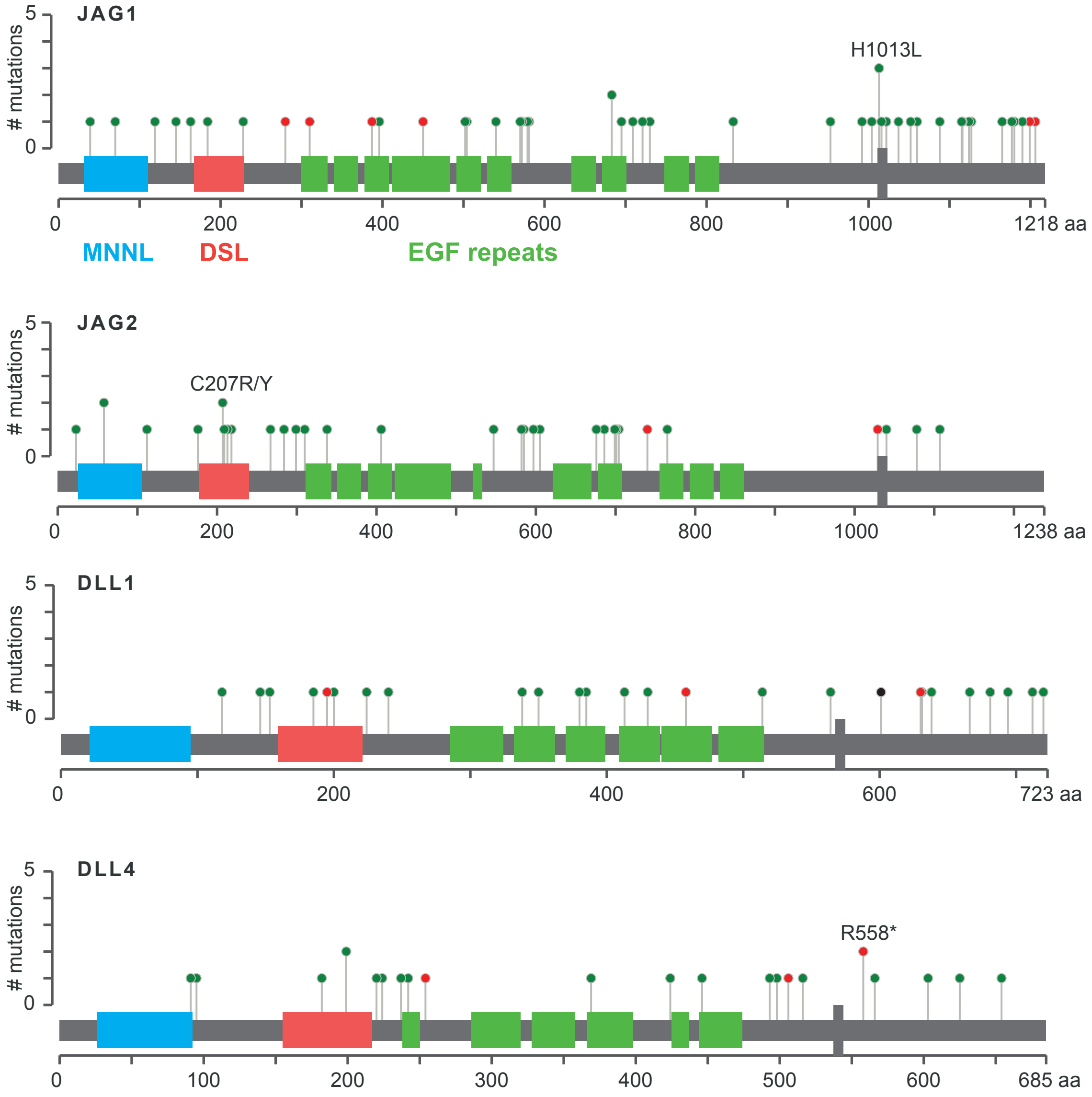

B

Mutational type and number of mutations for each Notch ligand					
#	Missense mutations	Frame shift alterations	Nonsense mutations	Indels	Splice Site SNP
JAG1	42	3	2	0	1
JAG2	30	1	0	0	1
DLL1	22	2	1	1	0
DLL4	19	1	3	0	0

Supplementary Figure 2

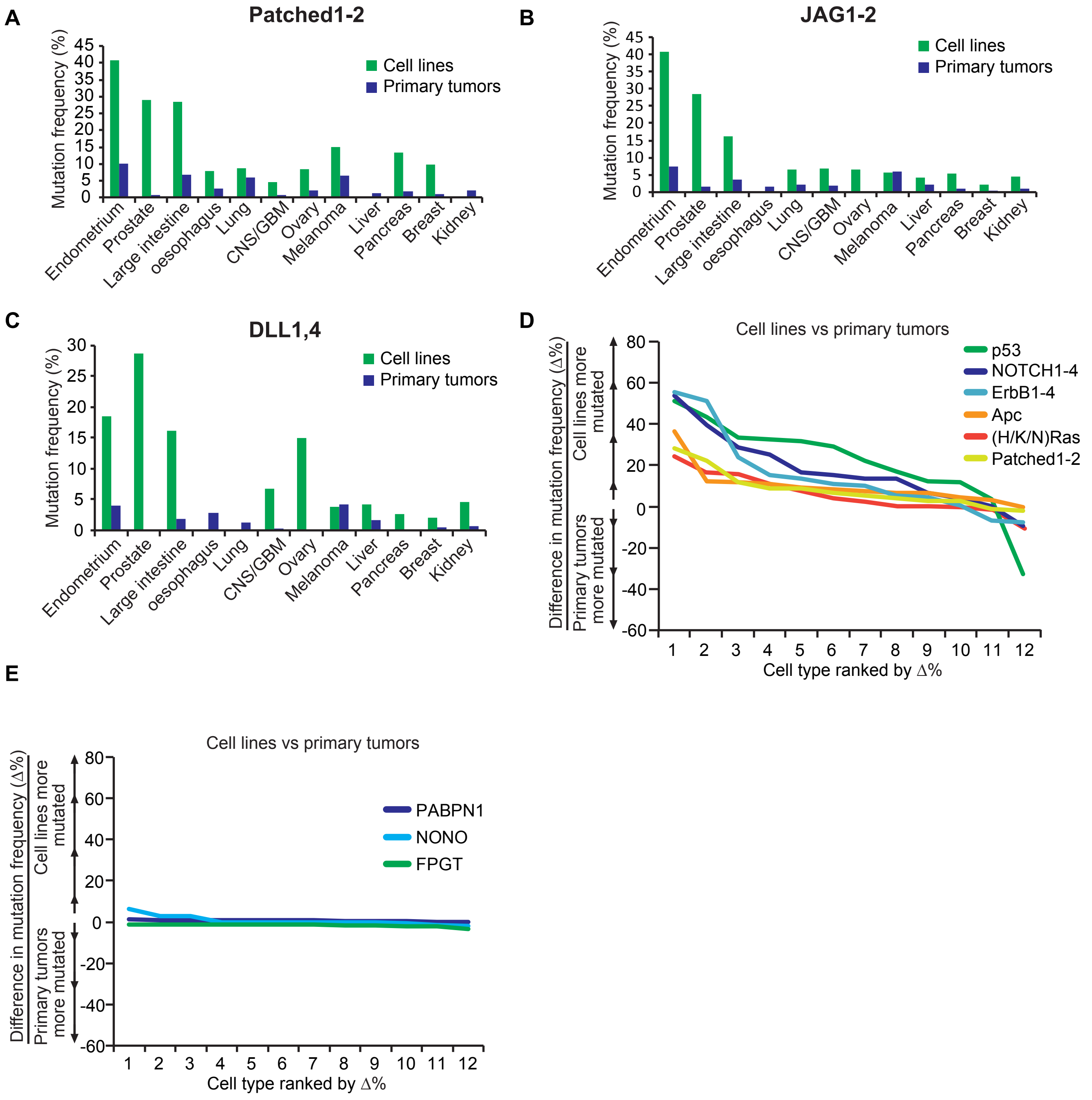

Supplementary Figure 3
